# Supplementary material for: Sample size calculation for a NanoString GeoMx spatial transcriptomics experiment to study predictors of fibrosis progression in non-alcoholic fatty liver disease
Source: Sci Rep. 2023 Jun 2;13:8943. doi: 10.1038/s41598-023-36187-0 (PMC10238473; doi:10.1038/s41598-023-36187-0)

Supplementary Figures

Sample size calculation for a NanoString GeoMx spatial transcriptomics experiment to study predictors of fibrosis progression in non-alcoholic fatty liver disease

Maria Ryaboshapkina[1+], Vian Azzu[2]

[1] Translational Science and Experimental Medicine, Research and Early Development, Cardiovascular, Renal and Metabolism (CVRM), BioPharmaceuticals R&D, AstraZeneca, Gothenburg, Sweden.

[2] Translational Science and Experimental Medicine, Research and Early Development, Cardiovascular, Renal and Metabolism (CVRM), BioPharmaceuticals R&D, AstraZeneca, Cambridge, UK.

[+]Corresponding author: maria.ryaboshapkina@astrazeneca.com

Fig. S1. Technical method comparison between linear regression on rlog counts and DESeq2 on raw counts. The plots show correlation between results obtained with both methods in NAFLD GSE135251. We randomly sampled 28 controls (F0) and 15 cases per more advanced fibrosis stage and evaluated scenarios where log2 fold changes were expected to be small **(a)**, medium **(b)** and large **(c and d)**. Fibrosis stages: F0 denotes no fibrosis, F1 minimal fibrosis, F2 moderate fibrosis, F3 advanced fibrosis and F4 cirrhosis. Each data point is a gene.

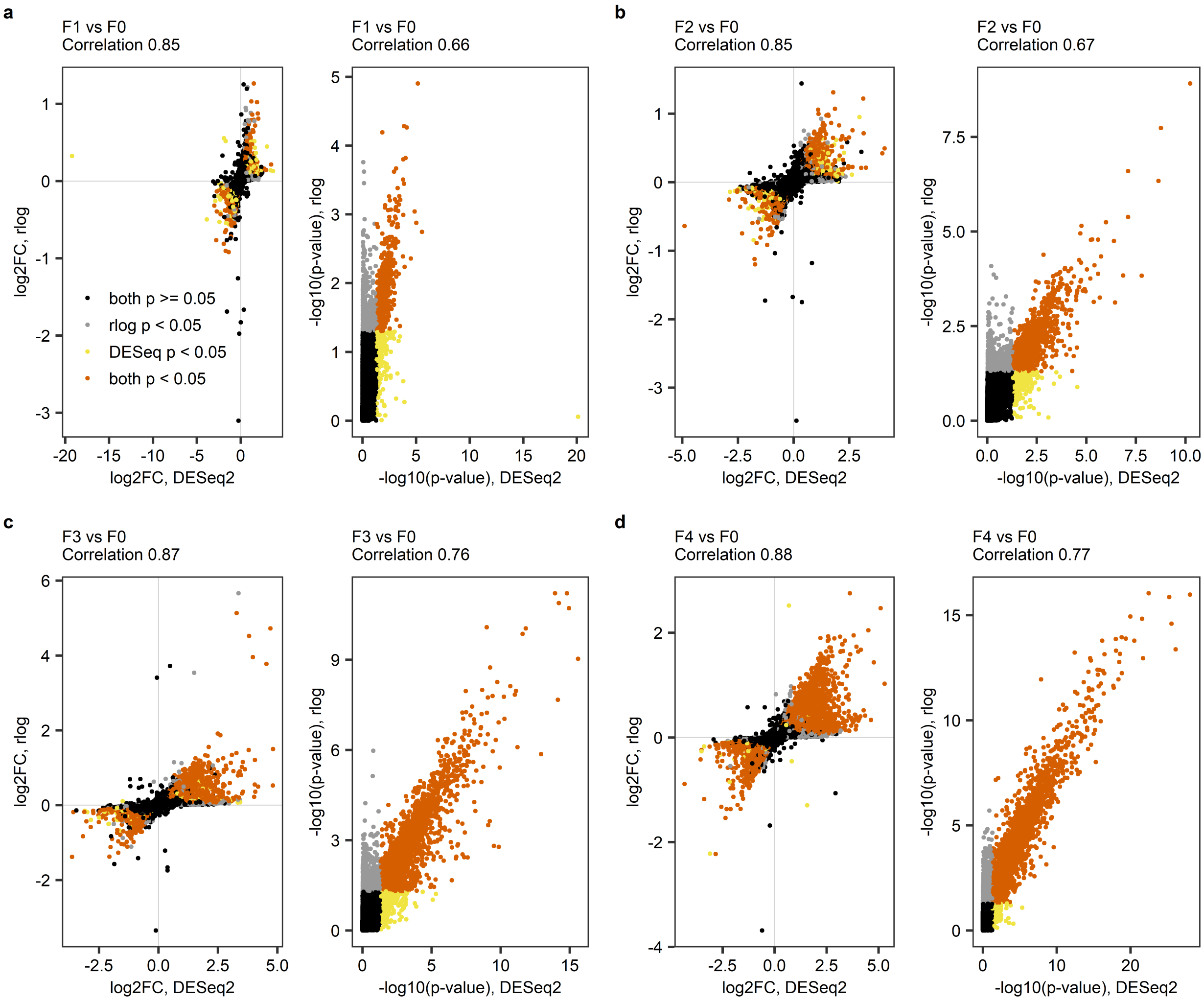

Fig. S2. Selection of a representative marker for the lipid metabolism score. (a) Top 10 out of the 44 lipid metabolism markers by similarity of their expression to the score, i.e., the normalized average expression of all 44 markers. **(b).** Relative expression abundance of these top 10 markers in hepatocytes and **(c)** enrichment in hepatocytes compared to fibrotic niche in Wu et al. normal samples adjacent to HCC.

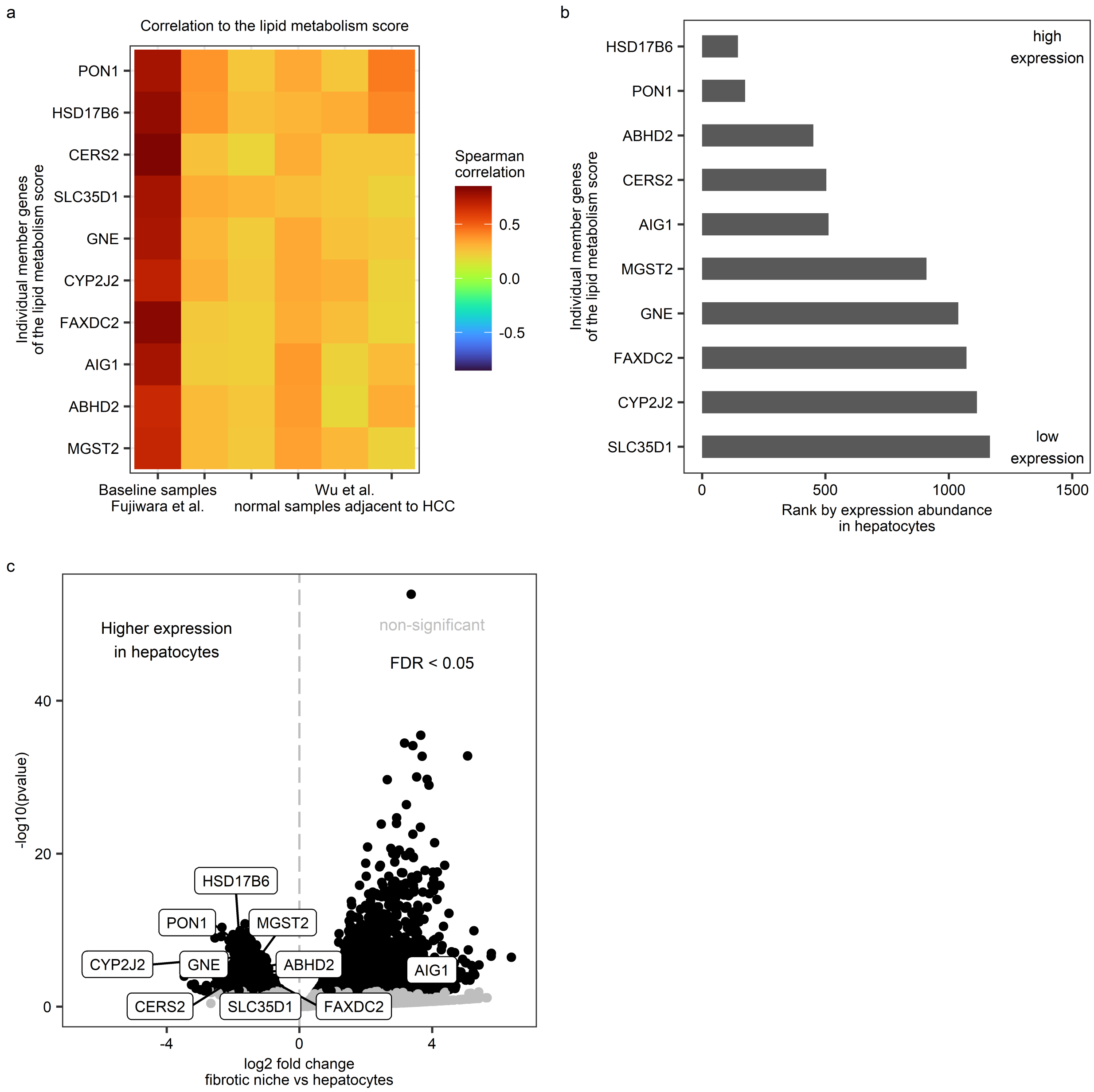

Fig. S3. Selection of a representative marker for the cytoskeleton score. (a) Top 10 out of the 89 cytoskeleton markers by similarity of their expression to the score, i.e., the normalized average expression of all 89 markers. **(b)**. Relative expression abundance of these top 10 markers in fibrotic niche and **(c)** enrichment in fibrotic niche compared to hepatocytes in Wu et al. normal samples adjacent to HCC.

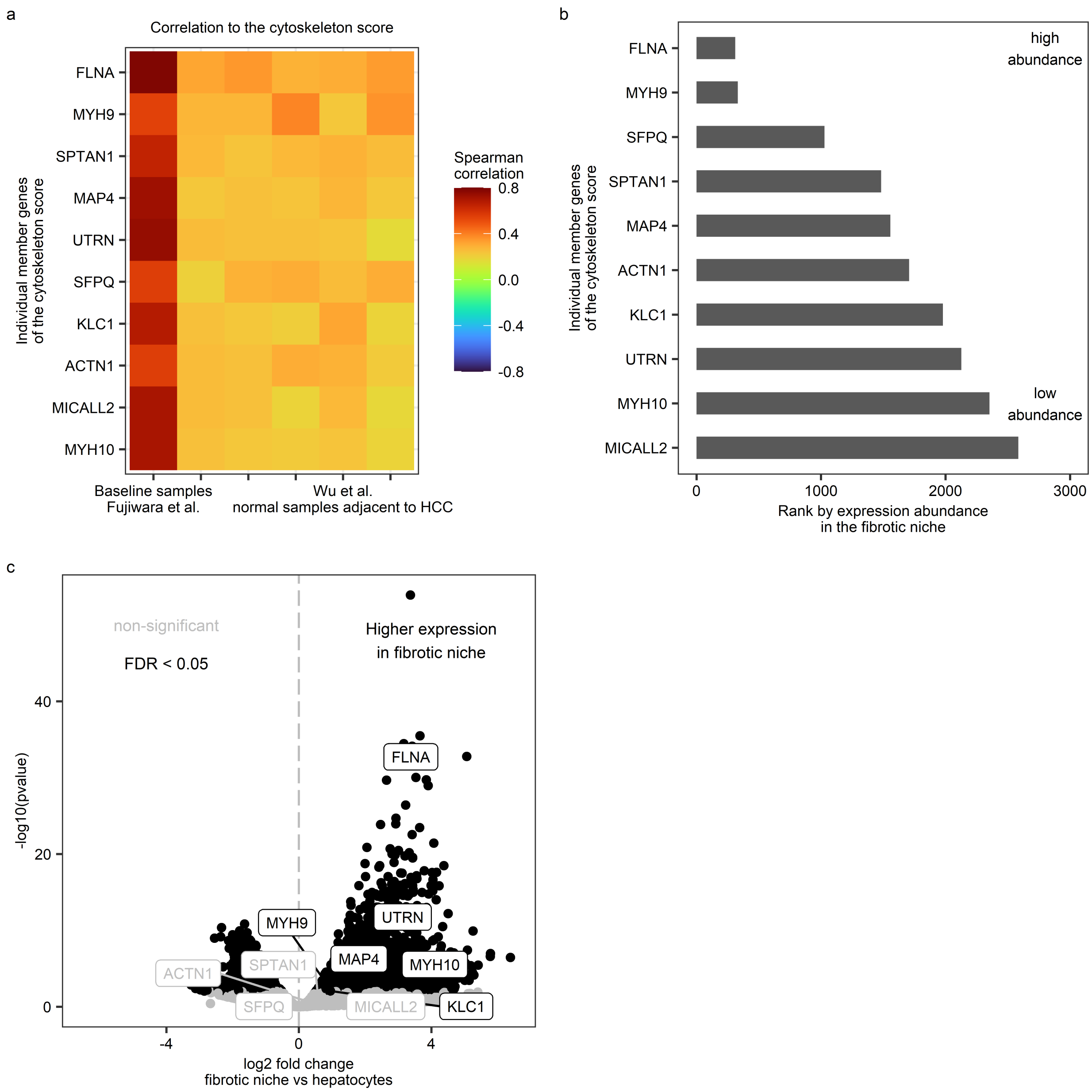

Fig. S4. Calibration curves. This analysis was conducted to re-scale expression fold changes from bulk liver to fibrotic niche **(a)** and hepatocyte fraction **(b)**. The smallest absolute log2 fold change between fibrosis progressors or fibrosis regressors and stable fibrosis individuals in bulk liver was taken to re-scale to the log2 fold change in the corresponding tissue fraction. Numbers on y axis indicate fractions of spiked-in counts. Log2 fold changes were estimated with DESeq2.

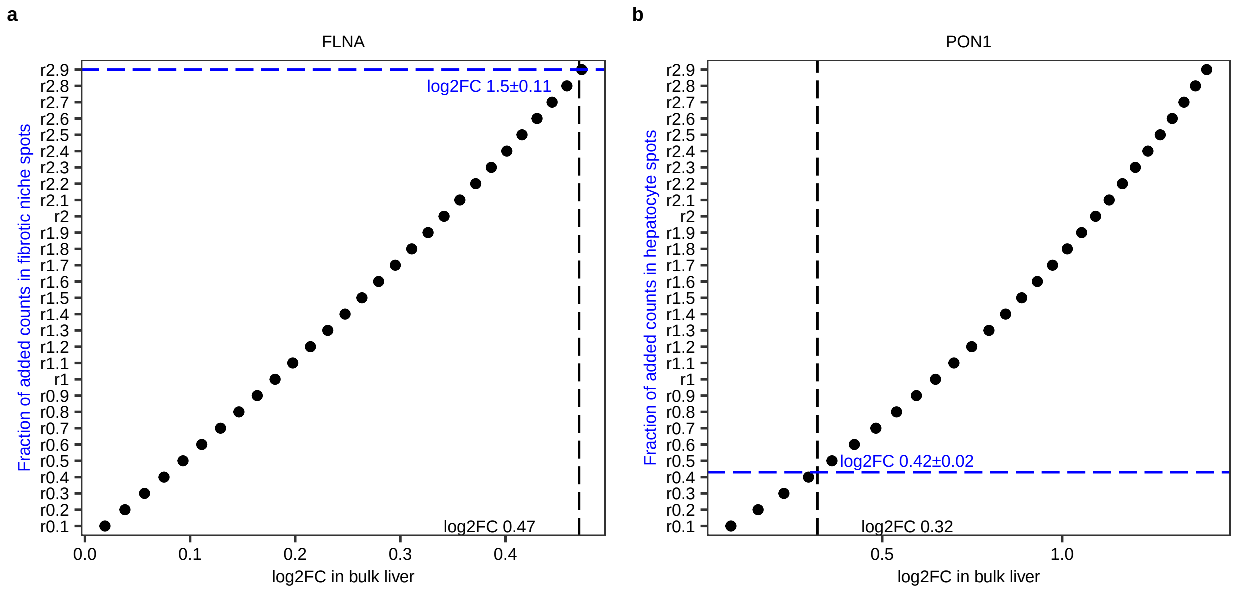

Fig. S5. Calibration curves in sensitivity analysis. This analysis was conducted to re-scale expression fold changes from bulk liver to fibrotic niche **(a)** and hepatocyte fraction **(b)**. The smallest absolute log2 fold change between fibrosis progressors or fibrosis regressors and stable fibrosis individuals in bulk liver was taken to re-scale to the log2 fold change in the corresponding tissue fraction. Numbers on y axis indicate fractions of spiked-in counts. Log2 fold changes were estimated with DESeq2.

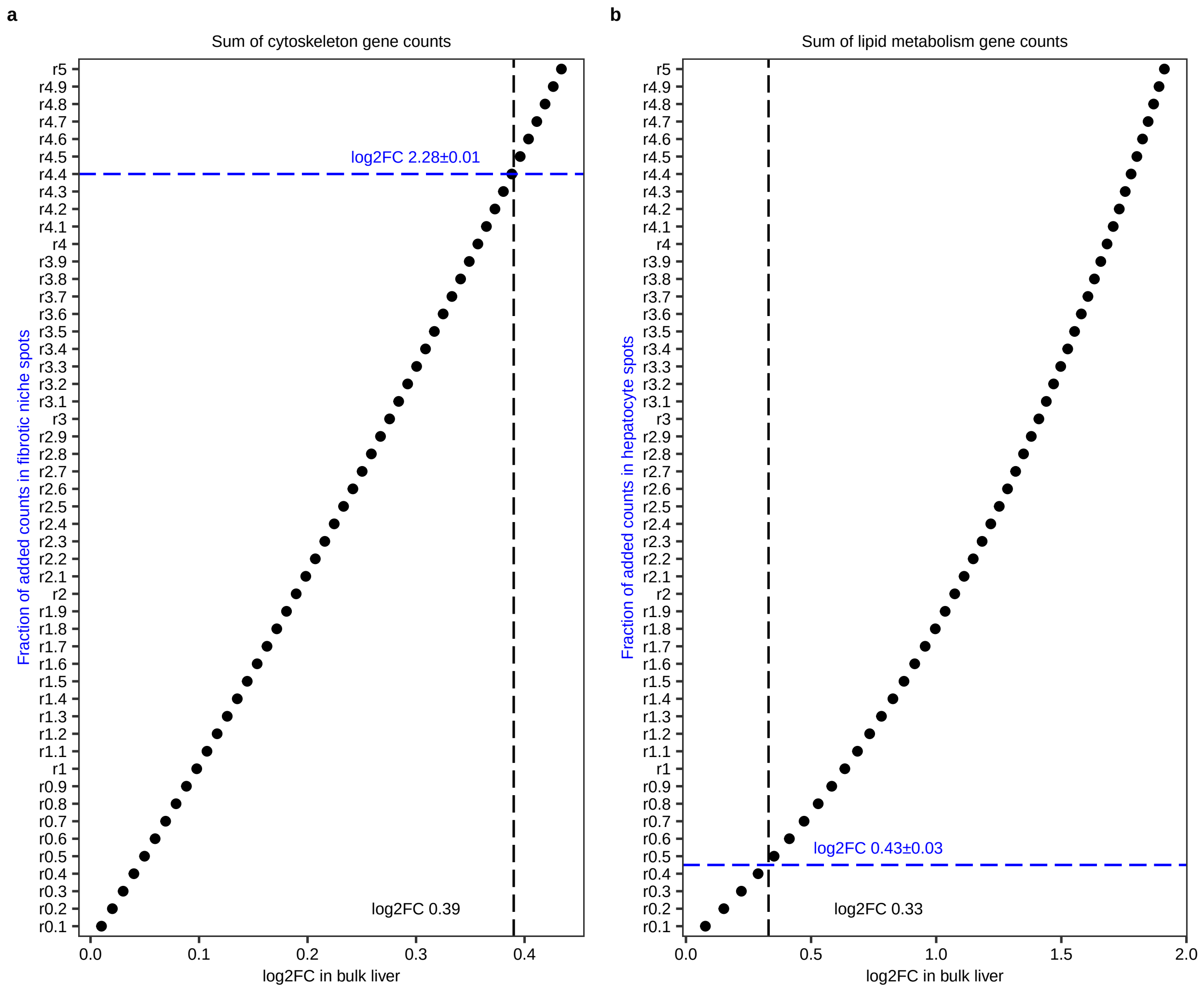

Supplement: Supplementary file 1 — Supplementary Figures. [file 41598_2023_36187_MOESM1_ESM.pdf]
